# Good Cooks

**Published:** 1874-06

**Authors:** 


### GOOD COOKS.

FIGURES a foot long would fail to convey to the mind of an average reader the amount of food destroyed by bad cooking and the diseases directly attributable to that cause. Cooks are kitchen made—without actual manipulation, without the use of the hands over the fire, a good cook was never made. Books give an idea of what ought to be done, and they tell how it ought to be done; and recipes are of great value, only if the reader takes the materials and mixes them and puts them on the fire. Besides having a good "receipt," a person must have a certain "knack" in following it out; and this is never properly done by a first trial, and seldom short of a half dozen. There must be practice; there is no substitute for that—intelligent practice. Look at bread making. How many cooks are there in a thousand who make a loaf of good wheat bread? On how many country tables in notable New England do we find good home made bread? A great pity is it that every mother does not feel it a matter of pride, and principle, and duty to instruct her daughters how to prepare food for the table, even though their fathers can leave each of them a million of money. A wife who knows how to prepare a cup of coffee or tea, make good bread, and broil a steak or a chicken as it ought to be done, and bake or boil a potato in the best manner, and knowing these, could show a servant and make her learn, or turn her out of doors, would be worth more to some men and their families than if they could speak a dozen modern languages, could sing divinely, and play beyond compare. Worth more, because she would add more to comfort, and enjoyment, and health, and happiness of the household. The best cooking school is home. It is the mother who should be the teacher of her daughter, and she should take it for granted at the outset that it can only be done in the kitchen; the child handling every ingredient, the mother never touching a thing, only give directions, and praise and encouragement, and what is more, would thus save money to struggling, and generous, and liberal husbands, while the sickness, and suffering, and disease prevented would be incomputable.

